# Evaluation of an online interactive Diabetes Needs Assessment Tool (DNAT) versus online self-directed learning: a randomised controlled trial

**DOI:** 10.1186/1472-6920-11-35

**Published:** 2011-06-16

**Authors:** Sara Schroter, Richard D Jenkins, Rebecca A Playle, Kieran M Walsh, Courtenay Probert, Thomas Kellner, Gerhard Arnhofer, David R Owens

**Affiliations:** 1 BMJ Editorial Office, BMJ Group, BMA House, Tavistock Square, London WC1H 9JR, UK; 2 BMJ onExamination, Cardiff Medicentre, Heath Park, Cardiff, CF14 4UJ, UK; 3 South East Wales Trials Unit, Department of Primary Care & Public Health, Cardiff University, 7th Floor, Neuadd Meirionnydd, Heath Park, Cardiff, CF14 4YS, UK; 4 BMJ Learning, BMJ Group, BMA House, Tavistock Square, London WC1H 9JR, UK; 5 AXDEV Europe, Ammerthalstr. 4 - 6, 85551 Kirchheim, Germany; 6 MSD, a subsidiary of Merck & Co. Corp., Hertford Road, Hoddesdon, EN11 9BU, UK; 7 Diabetes Research Unit, 1st Floor Academic Centre, University Hospital Llandough, Penlan Road, Penarth, CF64 2XX, UK

## Abstract

**Background:**

Methods for the dissemination, understanding and implementation of clinical guidelines need to be examined for their effectiveness to help doctors integrate guidelines into practice. The objective of this randomised controlled trial was to evaluate the effectiveness of an interactive online Diabetes Needs Assessment Tool (DNAT) (which constructs an e-learning curriculum based on individually identified knowledge gaps), compared with self-directed e-learning of diabetes guidelines.

**Methods:**

Health professionals were randomised to a 4-month learning period and either given access to diabetes learning modules alone (control group) or DNAT plus learning modules (intervention group). Participants completed knowledge tests before and after learning (primary outcome), and surveys to assess the acceptability of the learning and changes to clinical practice (secondary outcomes).

**Results:**

Sixty four percent (677/1054) of participants completed both knowledge tests. The proportion of nurses (5.4%) was too small for meaningful analysis so they were excluded. For the 650 doctors completing both tests, mean (SD) knowledge scores increased from 47.4% (12.6) to 66.8% (11.5) [intervention group (n = 321, 64%)] and 47.3% (12.9) to 67.8% (10.8) [control group (n = 329, 66%)], (ANCOVA p = 0.186). Both groups were satisfied with the usability and usefulness of the learning materials. Seventy seven percent (218/284) of the intervention group reported combining the DNAT with the recommended reading materials was "*very useful"/"useful"*. The majority in both groups (184/287, 64.1% intervention group and 206/299, 68.9% control group) [95% CI for the difference (-2.8 to 12.4)] reported integrating the learning into their clinical practice.

**Conclusions:**

Both groups experienced a similar and significant improvement in knowledge. The learning materials were acceptable and participants incorporated the acquired knowledge into practice.

**Trial registration:**

ISRCTN: ISRCTN67215088

## Background

Diabetes is an increasingly prevalent disease globally encompassing several specialties, thereby involving complex management regimes. Methods for the dissemination, understanding and implementation of available clinical guidelines need to be examined for their effectiveness. The evidence is limited, as reported in a systematic review[1] of various approaches to education for healthcare professionals, including the use of local consensus guidelines with combinations of reminders, audit and feedback[2-9]. Whilst there were improvements in the provision of care, patient outcomes were infrequently assessed. Indeed, most evaluative research of online Continuous Professional Development (CPD) focuses on participants' satisfaction and not on change in clinical practice or impact on patient and health outcomes[10]. Recent research in diabetes has focused on the mode of delivery of the education with mixed results[11,12].

Online learning for health professionals can be as effective as more traditional methods[10,13-15]. Online learning programs in diabetes have been shown to improve both guidelines knowledge[16,17] and compliance[18]. It can provide learners with the opportunity to personalise their learning, such as the order in which they read material, speed of their learning, and when and where they choose to learn[13]. With today's extensive choice of available learning opportunities, especially in electronic format, there is a need for a method to assist individuals with their professional development that is convenient and practical. Currently, CPD largely relies on healthcare professionals' self-perceived learning priorities[19]. However, evidence suggests this ability to self-assess is limited,[20] and even more so in the least competent[21]. Doctors have a tendency to select topics on which they already have some knowledge and not topics where they have a knowledge gap[20]. There are a number of methods to identify gaps in knowledge and clinical skills,[22] but not one definitive methodology, and using a variety of methods often provides a better overview[23]. Therefore, it may be of benefit to have some form of formal external needs assessment,[24] without exclusive reliance on it as this might discourage creativity and professionalism[19]. We describe a randomised controlled trial evaluating the effectiveness (in terms of knowledge gain, acceptability, practice change) of an interactive online Diabetes Needs Assessment Tool (DNAT) (which constructs an e-learning curriculum based on individually identified knowledge gaps), compared with self-directed e-learning of guidelines on diabetes.

## Methods

### Design

This study was designed to evaluate the effectiveness of an online interactive learning tool, the Diabetes Needs Assessment Tool (DNAT), in conjunction with a Learning Management System (LMS) to improve healthcare professionals' knowledge and competence to manage diabetes. It also evaluated the acceptability of this process of learning, and self-reported changes in clinical practice. The full study protocol has been published so we report a summarised version of the methods here[25].

### Participants

The Research Ethics Committee for Wales confirmed the study did not require full ethical review (personal communication 07 January 2009) and the research was carried out compliant with the Helsinki Declaration. Volunteers were recruited (between 20/02/09 and 01/04/09) through targeted emails to registered users of univadis^® ^(provider of online health care resources) and health professionals on the BMJ's contact database. Advertisements inviting English and German speaking practicing doctors and nurses to take part in an educational research project were placed in the BMJ, two German magazines (Der Hausarzt and Der Allgemeinarzt), and a newsletter (Aerztezeitung). Participants were required to be managing at least one person with diabetes per week and all participants consented to take part. In order to motivate participants to take part, they were informed that the time spent on learning activities for the project would contribute to their Continuing Professional Development (CPD) and that they would receive a personalised certificate of learning on completion of the course. They were also told that they would receive their test results along with the correct answers, and be given access to the most effective learning package at the end of the study free of charge. Additionally, those who completed all the assessments would be given a choice of access to one of three BMJ knowledge related products (English speaking participants) and some QUAIME AG online learning modules (German speaking participants).

### Randomisation, allocation concealment and blinding

Eligible registered participants on completion of Test 1 (described below) were randomised to either the control or intervention group. Optimal allocation with a ratio of 1:1 was used. Randomisation was balanced for language, ability (based on Test 1 score), doctor or nurse, years since qualification, and whether they were registered users of the webservice univadis^® ^and/or BMJ Learning, using a minimisation technique[25]. The total sample of health professionals recruited was divided into blocks of 24 and within each block a process of optimal allocation was undertaken. This involved obtaining all possible allocations and calculating a balance statistic[26]. One thousand allocations with the greatest degree of balance were identified and passed to an independent statistician within the South East Wales Trials Unit (SEWTU) at Cardiff University, who randomly selected a single allocation for each block. This was then returned to the trial statistician (RP) and the study database manager informed of the allocations.

All outcome measures were administered automatically online and all data held in the online database. Randomisation was carried out by an independent statistician and the coded allocations passed back to the DNAT data management team. The analysis was conducted by the trial statistician blinded to group allocation of participants.

### Interventions

All participants (both groups) were given access to use the same online Diabetes learning modules on an LMS (technical platform which tracks all user activities including which, how long and how often materials are used). All modules are applicable to European diabetes care practice and originated from BMJ Learning, Excerpta Medica, the International Diabetes Federation, and Elsevier Health Sciences. The modules include current evidence-based guidelines on diabetes, pre-diabetes and cardiovascular disease; important clinical areas and common difficulties in practice (Type 1, Type 2, diabetes in pregnancy and secondary causes of diabetes). The system hosting the learning modules was developed and hosted by univadis^®^. It was an unbranded ('white labelled') version of their website to focus learners on only the intended modules. Participants indicated which modules they completed and time spent using them.

#### Diabetes Needs Assessment Tool (DNAT)

In addition to having access to the Diabetes learning modules (described above), the intervention group were administered the DNAT, a new web-based interactive diabetes learning tool (developed by BMJ onExamination). Individuals' ability to self-assess learning needs is limited[20,21] so the aim of the DNAT is to identify individual learning needs and to indicate an appropriate learning source to meet those needs. The DNAT is a computerised adaptive test comprised of clinically rich case problems. The 253 test items had already been calibrated through use with a large number of learners. Items covered six categories: principles of diabetes, lifestyle, drug treatment, acute complications, microvascular and macrovascular complications. The DNAT can be completed over several sessions if required and takes the learner approximately 90 to 120 minutes to complete. Not all learners see the same number and type of questions as the test adapts to each individual's knowledge level. After each response the learner's ability is estimated based on their responses to all test items presented in a certain category. When an exit criterion is reached (e.g. probability of ability estimate or maximum test items answered) then the ability for that category is stored and the next category analysed. On completion of the DNAT, a personalised learning report is created matching learning needs with recommendations of the most appropriate Diabetes learning modules to meet those needs. At any stage a personalised report can be viewed listing the performance of the learner at that point.

### Primary outcome

#### Diabetes Knowledge Test

Two Diabetes Knowledge Tests containing 19 multiple choice questions were used, developed from the same large pool of calibrated items as the DNAT. Participants received an initial baseline test (Test 1) prior to randomisation and a further comparable test (Test 2) on completion of the learning period. The primary outcome was Test 2 percentage score.

### Secondary outcomes

An *Acceptability Survey *asked participants about the quality, relevance, presentation, perceived usefulness, and usability of the learning materials. Each was asked to indicate the extent to which they agreed/disagreed with a series of statements and respond to some open text questions. The intervention group was also asked specific questions about the acceptability of the DNAT including ease of use and usefulness.

A *Practice Change Survey *enquired if participants were able to integrate the learning into their clinical practice, about awareness of changes in their level of knowledge, competence and skills in managing people with diabetes, and requested specific examples of changes in their diabetes management as a result of the learning.

### Assessments and administration

All learning materials, assessments and surveys were administered to participants online at the study websites and all study communication was conducted by e-mail. There were two parallel websites, one for English speaking participants and the other for German speaking participants[25]. These independent websites hosted the DNAT, assessments and surveys and were managed by BMJ onExamination. At the start of the 4-month learning period participants were randomly allocated to their learning groups. Both groups received six automated reminders to use the materials and could choose which learning modules to access. Immediately after the learning period, access to materials was closed and participants were asked to complete Test 2 and an Acceptability Survey. After a further month, participants were asked to complete a Practice Change Survey. Participants were emailed up to 12 times reminding them to complete the knowledge test and surveys. Those who did not complete Test 2 and/or the Acceptability Survey were still asked to complete a Practice Change Survey.

### Statistical Analysis

A minimum sample size of 176 per group was estimated to have 80% power to detect an effect size of 0.3 at a significance level of 0.05. All analyses followed the intention to treat (ITT) principle and groups were analysed as randomised. Analysis of Covariance (ANCOVA) was used to compare Test 2 scores between groups adjusting for Test 1 scores as a covariate. Variables used in the balancing algorithm were also considered for inclusion as covariates. Secondary outcome analysis compared the survey outcomes between groups. Planned subgroup analyses involved the investigation of the learning outcomes within the language groups. In the first instance missing Test 2 data was assumed to be missing at random. Baseline checks were carried out to test this assumption. Missing Test 2 scores were assumed to have remained unchanged for the ITT analysis. A complete case analysis (CCA) was also carried out excluding those missing follow-up test scores.

## Results

### Participants

**Figure 1:**
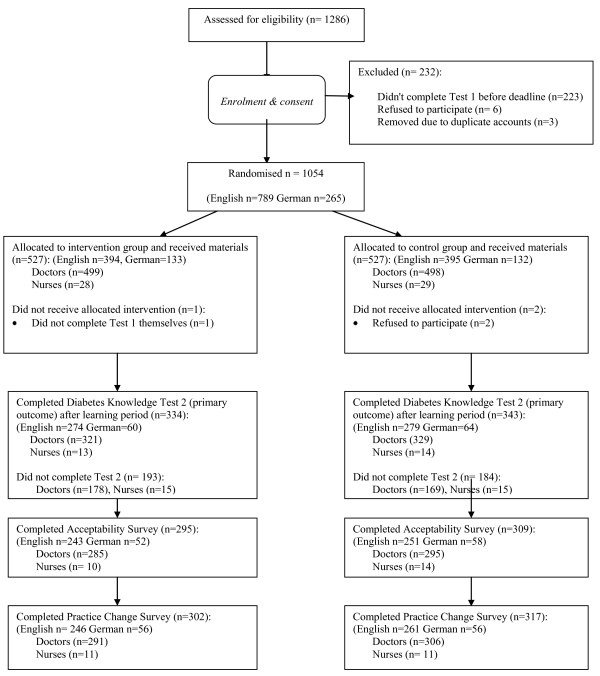
**Participant flow chart**.

Of 1286 participants assessed for inclusion, 232 did not meet inclusion criteria, leaving 1054 to be randomised (n = 789 English n = 265 German participants), Figure 1. A similar number of participants in each group did not complete Test 2 with 677 (64.2%) completing both knowledge tests and included in the CCA. Completers were more likely to be English than non-completers (81.7% vs. 62.0%), less likely to be registered univadis^® ^users (18.3% vs. 32.3%), but more likely to be registered for both BMJ learning and univadis^® ^(27.8% vs. 17.6). Table 1 shows baseline characteristics of the sample. The number of nurses recruited was small (n = 57), precluding inclusion in the analysis, with the remainder of the paper therefore restricted to the sample of doctors (n = 997).

**Table 1:** Baseline comparisons of intervention and control groups

Balancing Variable	Intervention Group	Control Group	Total
	n = 527	n = 527	n = 1054
Mean (SD) Baseline Knowledge Test Scores	46.7 (12.86)	46.7 (13.05)	46.7 (12.95)

Mean (SD) Years Post Qualification	15.9 (10.28)	16.3 (10.44)	16.1 (10.36)

Language, n (%)			

English	394 (74.8)	395 (75.0)	789 (74.9)

German	133 (25.2)	132 (25.0)	265 (25.1)

Health Professional Role, n (%)			

Doctor	499 (94.7)	498 (94.5)	997 (94.6)

Nurse	28 (5.3)	29 (5.5)	57 (5.4)

Registered users of univadis^® ^and/or BMJ Learning, n (%)			

None	137 (26.0)	138 (26.2)	275 (26.1)

univadis^®^	126 (23.9)	115 (21.8)	241 (22.9)

BMJ Learning	144 (27.3)	145 (27.5)	289 (27.4)

Both	120 (22.8)	129 (24.5)	249 (23.6)

### Primary outcome

The ITT analysis included all 997 doctors. Scores for those who did not complete Test 2 were assumed to remain unchanged from baseline. The mean knowledge test scores increased similarly in both groups: from 47.4% to 59.0% (n = 499) and 47.3% to 60.1% (n= 498) in the intervention and control groups, respectively.

The CCA used only those results from doctors who completed both knowledge tests (n = 650). Mean percentage test scores improved similarly in both groups: from 47.4% to 66.8%, n= 321 (intervention group) and from 47.3% to 67.8%, n= 329 (control group), Table 2. The ANCOVA showed no statistically significant difference between the groups for either ITT analysis (p = 0.172) or CCA analysis (p = 0.186), indicating no bias between groups in the CCA. The effect of the intervention is weaker as expected in the ITT analysis than the CCA, indicating the difference between a pragmatic result (effectiveness) and compliant result (efficacy) of approximately 8 percentage points in test scores.

**Table 2:** Primary analysis of knowledge test scores

	Baseline score % (SD)	Follow-up score (ITT) % (SD)	Follow-up score (CCA) % (SD)	Mean Difference (CCA-baseline) [95% CI]	ANCOVA* (CCA) p-value
Intervention group	47.4 (12.6)	59.0 (15.8) N = 499	66.8 (11.5) N = 321	18.1 [16.6 to 19.6]	

Control group	47.3 (12.9)	60. 1 (15.9) N = 498	67.8 (10.8) N = 329	19.4 [18.0 to 20.8]	

Total	47.3 (12.8)	59.6 (15.8) N = 997	67.3 (11.1) N = 650	18.7 [17.7 to 19.8]	0.186

* ANCOVA includes baseline score as covariate

SD: standard deviation

ITT: Intention to treat analysis

CCA: Complete case analysis

CI: Confidence interval

The only significant covariate was language where test scores were lower at baseline for German participants but improved by an equivalent amount during the study.

### Secondary outcomes

Five hundred and eighty participants completed the Acceptability Survey (n = 285 intervention group, n = 295 control group). Both groups were very satisfied with the usefulness and usability of the learning materials (Table 3). In addition, 76.8% (218/284) of the intervention group reported that it was "*very useful*"/"*useful*" to combine the recommendations from the needs assessment tool with the reading materials.

**Table 3:** Doctors responses to key questions on the Acceptability and Practice Change surveys by study group

	N (%) Strongly agree + agree Intervention Group	N (%) Strongly agree + agree Control Group	Difference (95% CI)
**ACCEPTABILITY SURVEY**			

The learning materials have improved my overall understanding of the management of diabetes	237/285 (83.1)	251/295 (85.1)	-1.9 (-7.9 to 4.0)
The learning materials covered important subjects/areas	244/285 (85.6)	269/294 (91.5)	-5.9 (-11.2 to -0.7)
Locating relevant content in the learning materials was easy	189/285 (66.3)	187293 (63.9)	2.5 (-5.3 to 10.2)
The learning materials were relevant for my professional development	243/285 (85.3)	258/294 (87.8)	-2.5 (-8.1 to 3.1)
The learning materials helped me to achieve my personal learning objectives	183/285 (64.2)	195/295 (66.1)	-1.9 (-9.6 to 5.8)
The learning materials have made me aware of my potential knowledge gaps/learning needs	257/285 (90.1)	260/295(88.1)	2.0 (-3.1 to 7.2)
The learning experience has changed/will change how I will treat patients with diabetes under certain circumstances	220/285 (77.2)	224/295 (75.9)	1.3 (-5.6 to 8.1)
The learning materials have helped me to improve my skills in diabetes management	216/285 (75.8)	227/295 (77.0)	-1.2 (-8.1 to 5.7)
The learning materials have made me aware of the potential gaps in my practical skills	213/285 (74.7)	220/295 (74.6)	0.2 (-6.9 to 7.2)
What I have learnt will influence my professional practice	240/285 (84.2)	251/294 (85.4)	-1.2 (-7.1 to 4.7)
Needs assessment tool was easy to use	233/284 (82.0)	NA	NA
Needs assessment tool helped to identify my learning needs	207/284 (72.9)	NA	NA
Needs assessment tool helped to identify skills I needed to improve	191/284 (67.3)	NA	NA
Needs assessment tool gave me suggestions for further reading in areas that were important to me	194/284 (68.3)	NA	NA
Needs assessment tool saved me time identifying my personal learning needs	161/284 (56.7)	NA	NA

**PRACTICE CHANGE SURVEY**			

	N (%) reporting improvement Intervention Group	N (%) reporting improvement Control Group	Difference (95% CI)

Awareness of change in your level of COMPETENCE in managing your patients with diabetes since undertaking this learning experience	217/291 (74.6)	229/306 (74.8)	-11.3 (-17.8 to -4.8)
Awareness of change in your level of clinical SKILLS in managing your patients with diabetes since undertaking this learning experience	187/291 (64.3)	205/306 (67.0)	-5.7 (-11.0 to -0.5)
Awareness of change in your level of KNOWLEDGE about how to manage patients with diabetes since undertaking this learning experience	249/291 (85.6)	267/306 (87.3)	-1.0 (-8.5 to 6.5)

Five hundred and ninety seven participants completed the Practice Change Survey. There was no difference between the groups in ability to integrate the learning into clinical practice (intervention group: 184/287 (64.1%), control group: 206/299 (68.9%); 95% CI for the difference (-2.8 to 12.4)). The majority of both groups reported improvement in their competence, skills and knowledge in managing people with diabetes since undertaking the learning (Table 3).

### Additional unplanned analysis

Doctors were asked how many learning modules they had accessed. Of the 532 who responded, those in the intervention group (n = 235) accessed fewer modules [median = 6 (IQR 3, 9)] than those in the control group (n = 297) [median = 8 (IQR 3, 14), with the same overall knowledge gain.

## Discussion

The use of an online interactive learning tool for diabetes did not result in significantly greater knowledge gain than administration of high quality learning materials alone. Both groups showed significant knowledge improvement and a high proportion reported integrating this knowledge into practice confirming that online CPD learning for doctors can be effective[15]. There was no difference between the groups in terms of the acceptability of the learning experience. The majority of the intervention group were very satisfied, reporting that the DNAT was easy to use and a useful addition to the learning modules.

There is only limited research to evaluate the effectiveness of different methods of delivering online learning to doctors. The majority of studies have compared some formats of online learning against traditional face-to-face or didactic learning demonstrating that online learning can be effective at improving knowledge and changing clinical practice[15]. We report here the first study to assess the effectiveness of an online interactive needs assessment tool on knowledge acquisition and implementation into clinical practice. Diabetes is a complex disease and necessitates a multifactorial approach to management. There is a multitude of resources available on the topic of diabetes. The needs assessment tool was therefore designed to help focus the learner on their areas of weakness (e.g. the role of new therapeutic agents, initiation, and optimisation of insulin therapy and/or the management of the diabetic foot), helping them to chose from the many learning resources available. Studies have also shown that health professionals are poor at identifying their own learning needs,[20] especially those with the greatest need[21]. We hypothesised that an interactive tool informing learners of their learning needs and giving access to appropriate learning modules could be an effective method of learning over and above just providing them with the same resources and allowing them to identify their own needs. We also allowed all participants to choose which modules they wanted to study given concern over the exclusive reliance on needs assessment tools[19]. The fact that we found no significant difference between the two learning groups may have been due to the fact that both groups had access to the same high quality learning resources. Learners in the intervention group were not provided with correct answers to the questions in the needs assessment tool during the learning period and this may have lessened the impact of the DNAT.

We recruited over a thousand participants in less than 6 weeks and who remained motivated throughout the study. This clearly demonstrates that there is a great demand amongst clinicians for good quality educational materials on diabetes. Knowledge, practice change and acceptability were also assessed in contrast to many studies which focus on just participant satisfaction[10]. Our study also compared two different formats of online learning rather than simply comparing the intervention against a control group with no resources, where we would have likely detected a group difference.

Limitations of the study include the self-selected and highly motivated sample. At the start of the study, the majority of the participants were already registered users of online learning resources (BMJ or univadis^®^) and may prefer online learning. Also, practice change was assessed in the short term and based on self-reporting rather than observation. However, over two-thirds of the sample self-reported that they had integrated their learning into clinical practice and many were able to substantiate this with specific examples. The next stage in CPD research should be to see whether online learning can result in improvements to the health status of the target population[22], although this approach is not without challenges. Whilst we conducted the study with two language groups, we do not know if the results are generalisable to other language groups. As would be expected of a trial of this nature involving busy health professionals, many participants dropped out over time. However, attrition rates were similar in both groups and more than 60% completed the primary outcome in each group. The optimal period for a learning intervention is not known. It is possible that the 4 month period was too long and caused some participants to drop out, but it needed to be long enough for learners to consolidate their learning. Our high recruitment rate when participants were informed of the study expectations suggests that the study period and study expectations were not unreasonable.

We did not pre-specify a comparison between groups in terms of efficiency (number of courses accessed) so the findings should be interpreted with caution. However, it is interesting to observe that the intervention group accessed fewer learning modules while achieving a similar level of knowledge improvement suggesting that the DNAT may demonstrate a more efficient route of knowledge acquisition. Further research in assessing the efficiency of the DNAT is required.

## Conclusions

Doctors need to stay up to date with current guidelines and online learning offers the opportunity for doctors to learn in a flexible and convenient style around busy schedules. This study demonstrated that online learning for doctors in the field of diabetes is acceptable and effective in terms of improving knowledge and changing clinical practice. We did not find a significant difference in outcomes between those that used the online interactive learning tool and those that did not, despite the learners reporting that the tool was easy to use and useful. Whilst this interactive learning tool did not improve knowledge over and above self-directed learning, an improved tool might be more effective and efficiency might be a more appropriate primary outcome for further research.

## Abbreviations

DNAT: Diabetes Needs Assessment Tool; BMJ Group: BMJ Publishing Group; SEWTU: South East Wales Trials Unit, Cardiff University

## Competing interests

SS is a full time employee of the BMJ Group. She regularly conducts research into all aspects of publishing for BMJ Group and will not benefit financially from the outcome of this study.

RDJ is a Director of BMJ onExamination. He is the Principle Investigator for the study and developed the DNAT and diabetes knowledge tests from test items generated by BMJ onExamination. He will not benefit financially from the success of the DNAT.

RP: No competing interests.

KW is editor of BMJ Learning and works for the BMJ Group. He is paid a fixed salary.

CP was Chief Information Officer for BMJ onExamination. He led the team that developed the websites for the study. He will not benefit financially from the success of the DNAT. All of the technological work in developing the DNAT tool was carried out at BMJ onExamination and is entirely independent of any other organisation.

At the time of the research, TK was a full time employee of MSD. TK now works for AXDEV Europe. He helped design the study and helped with the marketing campaign to recruit potential participants. He did not have access to the data until after it was analysed by researchers at Cardiff University and then was only given access to anonymised data.

GA is a full time employee of MSD. He helped design the study and helped with the set up of the learning management system and the provision of the online learning modules. He did not have access to the data until after it was analysed by researchers at Cardiff University and then was only given access to anonymised data.

DO has conducted lectures, nationally and internationally and served as a consultant for a number of pharmaceutical companies for which he has received honoraria. He will not benefit financially from the success of the DNAT.

## Authors' contributions

SS formulated the research question, designed the study, helped with trial management and interpretation of the results, and wrote the first draft of this manuscript. RDJ is Principal Investigator and joint guarantor of this paper with SS. RDJ developed the DNAT and knowledge tests, and helped with formulating the research question, study design, interpretation of the results and revising the manuscript. CP was Chief Information Officer at onExamination and led the software design team that enabled the DNAT to run on the onExamination platform and project managed the study design and helped revise the manuscript. RP contributed to the study design, formulated the statistical analysis plan, independently conducted the statistical analysis, and helped revise this manuscript. KW contributed to the study design and helped revise this manuscript. TK contributed to the study design, managed the recruitment plan for the German participants, and helped revise this manuscript. GA contributed to the study design, helped in the technical provision of the learning interventions and the learning management system, and helped revise the manuscript. DO contributed to the study design, interpretation of the results, and helped revise the manuscript. All authors read and approved the final manuscript.

## Pre-publication history

The pre-publication history for this paper can be accessed here:

http://www.biomedcentral.com/1472-6920/11/35/prepub

## References

[B1] Renders CM Valk GD Griffin SJ Interventions to improve the management of diabetes in primary care, outpatient, and community settings: a systematic review Diabetes Care 2001 24 10 1821 33 10.2337/diacare.24.10.1821 11574449

[B2] Lobach DF Hammond WE Development and evaluation of a Computer-Assisted Management Protocol (CAMP): improved compliance with care guidelines for diabetes mellitus Proc Annu Symp Comput Appl Med Care 1994 787 91 PMC2247833 7950032

[B3] Carlson A Rosenqvist U Diabetes care organization, process, and patient outcomes: effects of a diabetes control program Diabetes Educ 1991 17 1 42 8 10.1177/014572179101700109 1986903

[B4] Feder G Griffiths C Highton C Do clinical guidelines introduced with practice based education improve care of asthmatic and diabetic patients? A randomised controlled trial in general practices in east London BMJ 1995 311 1473 8 8520339 10.1136/bmj.311.7018.1473 PMC2543702

[B5] Ward A Kamien M Mansfield F Educational feedback in the management of type 2 diabetes in general practice Education for General Practice 1996 7 142 50

[B6] Mazzuca SA Vinicor F Einterz RM Effects of the Clinical Environment on Physicians' Response to Postgraduate Medical Education American Educational Research Journal 1990 27 3 473 88

[B7] Benjamin EM Schneider MS Hinchey KT Implementing practice guidelines for diabetes care using problem-based learning. A prospective controlled trial using firm systems Diabetes Care 1999 22 10 1672 8 10.2337/diacare.22.10.1672 10526733

[B8] Litzelman DK Slemenda CW Langefeld CD Reduction of lower extremity clinical abnormalities in patients with non-insulin-dependent diabetes mellitus Ann Intern Med 1993 119 1 36 41 A randomized, controlled trial. 8498761 10.7326/0003-4819-119-1-199307010-00006

[B9] Mazze RS Etzwiler DD Strock E Staged diabetes management. Toward an integrated model of diabetes care Diabetes Care 1994 17 Suppl 1 56 66 8088226

[B10] Curran VR Fleet L A review of evaluation outcomes of Web-based continuing medical education Med Educ 2005 39 6 561 7 10.1111/j.1365-2929.2005.02173.x 15910431

[B11] Gerstein HC Redd SSK Dawson KG controlled evaluation of a national continuing medical education programme designed to improve family physicians' implementation of diabetes-specific clinical practice guidelines Diabetic Medicine 2001 16 11 964 9 10.1046/j.1464-5491.1999.00159.x 10588528

[B12] Perria C Mandolini D Guerrera C Implementing a guideline for the treatment of type 2 diabetics: results of a Cluster- Randomized Controlled Trial (C-RCT) BMC Health Serv Res 2007 7 79 10.1186/1472-6963-7-79 17547760 PMC1904445

[B13] Ruiz JG Mintzer MJ Leipzig RM The Impact of e-learning in medical education Acad Med 2006 81 3 207 12 10.1097/00001888-200603000-00002 16501260

[B14] Wutoh R Boren SA Balas EA eLearning: a review of Internet-based continuing medical education J Contin Educ Health Prof 2004 24 1 20 30 10.1002/chp.1340240105 15069909

[B15] Cook DA Levinson AJ Garside S Internet-based learning in the health professions: a meta-analysis JAMA 2008 300 10 1181 96 10.1001/jama.300.10.1181 18780847

[B16] Stewart M Marshall JN Østbye T Effectiveness of case-based on-line learning of evidence-based practice guidelines Fam Med 2005 37 2 131 8 15690254

[B17] Wiecha JM Chetty VK Pollard T Web-based versus face-to-face learning of diabetes management: the results of a comparative trial of educational methods Fam Med 2006 38 9 647 52 17009189

[B18] Weston CM Sciamanna CN Nash DB Evaluating online continuing medical education seminars: evidence for improving clinical practices Am J Med Qua 2008 23 6 475 83 10.1177/1062860608325266 19001103

[B19] Grant J Learning needs assessment: assessing the need BMJ 2002 324 156 9 10.1136/bmj.324.7330.156 11799035 PMC64520

[B20] Sibley JC Sackett DL Neufeld V A randomized trial of continuing medical education N Engl J Med 1982 306 9 511 5 10.1056/NEJM198203043060904 7057858

[B21] Colthart I Bagnall G Evans A The effectiveness of self-assessment on the identification of learner needs, learner activity, and impact on clinical practice: BEME Guide no. 10 Medical Teacher 2008 30 2 124 45 10.1080/01421590701881699 18464136

[B22] Moore DE Jr Green JS Gallis HA Achieving desired results and improved outcomes: integrating planning and assessment throughout learning activities J Contin Educ Health Prof 2009 29 1 1 15 10.1002/chp.20001 19288562

[B23] Walsh K How to assess your learning needs J R Soc Med 2006 99 29 31 10.1258/jrsm.99.1.29 16388053 PMC1325078

[B24] Davis DA Mazmanian PE Fordis M Accuracy of Physician Self-assessment Compared With Observed Measures of Competence: A Systematic Review JAMA 2006 296 9 1094 1102 10.1001/jama.296.9.1094 16954489

[B25] Schroter S Jenkins D Playle R Evaluation of an online Diabetes Needs Assessment Tool (DNAT) for health professionals: a randomised controlled trial Trials 2009 10 63 10.1186/1745-6215-10-63 19642984 PMC3224950

[B26] Carter BA Hood K Balance algorithm for cluster randomized trials BMC Med Res Methodol 2008 8 65 10.1186/1471-2288-8-65 18844993 PMC2588445

